# Acute kidney injury in patients with severe COVID-19 in Mexico

**DOI:** 10.1371/journal.pone.0246595

**Published:** 2021-02-08

**Authors:** Gustavo A. Casas-Aparicio, Isabel León-Rodríguez, Claudia Alvarado-de la Barrera, Mauricio González-Navarro, Amy B. Peralta-Prado, Yara Luna-Villalobos, Alejandro Velasco-Morales, Natalia Calderón-Dávila, Christopher E. Ormsby, Santiago Ávila-Ríos

**Affiliations:** 1 Centro de Investigación en Enfermedades Infecciosas, Instituto Nacional de Enfermedades Respiratorias Ismael Cosío Villegas, Mexico City, Mexico; 2 Resident Doctor at Instituto Nacional de Enfermedades Respiratorias Ismael Cosío Villegas, Mexico City, Mexico; University of Sao Paulo Medical School, BRAZIL

## Abstract

**Introduction:**

Some patients with COVID-19 pneumonia present systemic disease involving multiple systems. There is limited information about the clinical characteristics and events leading to acute kidney injury (AKI). We described the factors associated with the development of AKI and explored the relation of AKI and mortality in Mexican population with severe COVID-19.

**Methods:**

We retrospectively reviewed the medical records of individuals with severe pneumonia caused by SARS-CoV-2 hospitalized at the largest third-level reference institution for COVID-19 care in Mexico between March and April 2020. Demographic information, comorbidities, clinical and laboratory data, dates of invasive mechanical ventilation (IMV) and hospitalization, mechanical-ventilator settings and use of vasoactive drugs were recorded.

**Results:**

Of 99 patients studied, 58 developed AKI (58.6%). The risk factors for AKI were older age (OR = 1.07, 95% CI = 1.01–1.13, p = 0.024); obesity (OR = 6.58, 95% CI = 1.8–24.05, p = 0.040); and the need for IMV (OR = 6.18, CI = 1.29–29.58, p = 0.023). The risk factors for mortality were obesity (OR = 5.57, 95% CI = 1.48–20.93, p = 0.011); requirement of vasoactive drugs on admission (OR = 5.35, 95% CI = 1.16–24.61, p = 0.031); and AKI (OR = 8.61, 95% CI = 2.24–33.1, p = 0.002). In-hospital mortality was significantly higher in patients with AKI stage 3 (79.3%) and AKI stage 2 (68.7%) compared with those with AKI stage 1 (25%; p = 0.004). Fifty-three patients underwent the furosemide stress test (FST) to predict progression to AKI stage 3. Of those, 12 progressed to AKI stage 3 (22%). The ROC curve for the FST had an AUC of 0.681 (p = 0.009); a sensitivity of 81.6% and a specificity of 54.5%.

**Conclusions:**

AKI was common in our cohort of patients with severe pneumonia caused by SARS-CoV-2 infection. The risk factors for AKI were older age, obesity and the need for of IMV on admission. The risk factors for mortality were obesity, requirement of vasoactive drugs on admission and AKI. Mortality was more frequent in patients with AKI stages 2–3. The FST had an acceptable predictive capacity to identify patients progressing to AKI stage 3.

## Introduction

In December 2019, a series of pneumonia cases of unknown cause emerged in Wuhan, Hubei Province, China, with clinical presentations resembling viral pneumonia [1]. The pneumonia spread quickly to other provinces of China and overseas. A novel coronavirus was identified by the Chinese Center for Disease Control and Prevention from the throat swab sample of a patient and was provisionally named 2019-nCoV by the World Health Organization (WHO) [2]. Based on phylogeny, taxonomy and established practice, the International Committee on Taxonomy of Viruses renamed the virus as Severe acute respiratory syndrome coronavirus-2 (SARS-CoV-2) [3]. WHO subsequently declared coronavirus disease 2019 (COVID-19) a public health emergency of international concern [4]. COVID-19 is primarily manifested as a respiratory tract infection, but emerging data indicate that it should be regarded as a systemic disease involving multiple systems, including cardiovascular, respiratory, gastrointestinal, neurological, hematopoietic, immune and renal [5–8]. Of note, after lung infection, the virus may enter the blood, accumulate in the kidney and cause damage to resident renal cells, with a significantly higher risk for in-hospital death [8]. Thus, understanding how the kidney is affected by SARS-CoV-2 is particularly relevant. The incidence of acute kidney injury (AKI) in hospitalized patients with COVID-19 varies across populations, but a large multicenter retrospective cohort study in New York reported AKI in 37% of hospitalized patients, and 35% of those died [9]. AKI initiation coincides with the development of Acute Respiratory Distress Syndrome (ARDS), and these alterations are typical of patients progressing to the most severe stage of illness involving extra-pulmonary systemic hyperinflammation [10].

The National Institute of Respiratory Diseases (INER) is the largest third-level national referral center for COVID-19 in Mexico City. Since early January 2020, this institution was gradually repurposed for the treatment of patients with COVID-19 exclusively. Since February 28, 2020, when the first Mexican patient was diagnosed with COVID-19, a high proportion of critically ill patients have been admitted at the INER. The aim of this study was to describe the factors associated with the development of AKI and explore the relation of AKI and mortality in the Mexican population with severe COVID-19.

## Methods

### Study population

The study was conducted at the INER, the largest third-level institution designated by the Mexican Government for COVID-19 care. All medical records of individuals with severe pneumonia caused by SARS-CoV-2 hospitalized at the INER between March and April 2020 were retrospectively reviewed. The Institutional Review Board (Comité de Ética en Investigación and Comité de Investigación del INER) approved the study and waived the requirement for informed consent due to the retrospective design of the study (Approval No. C39-20). Data were fully anonymized before being accessed. We included individuals with diagnosis of severe pneumonia caused by SARS-CoV-2, confirmed by real-time reverse transcription–polymerase chain reaction (rRT-PCR); 18 years of age or older; with no history of chronic kidney disease (CKD), as indicated by kidney ultrasound and direct interrogation of patients about CKD medical history; and ratio of partial arterial oxygen pressure/inspired oxygen fraction (PaO_2_/FiO_2_) <300 mm Hg on admission. Pregnant women were not included in the study. Patients with incomplete clinical records were excluded. Patients with SARS-CoV-2 severe pneumonia were defined as those with clinical data of respiratory distress, bilateral alveolar opacities in 2 or more lobes, a ratio of PaO_2_/FiO_2_ < 300 mm Hg and a positive result for SARS-CoV-2-rRT-PCR assay [11] in nasopharyngeal swab. Prone ventilation was used for the treatment of ARDS as a strategy to improve oxygenation when traditional modes of ventilation failed.

The primary outcome was the development of AKI. The secondary outcome was 30-day mortality in the group with AKI and the group without AKI. Recorded variables included demographic and anthropometric variables, symptoms, comorbidities, treatments, critical care variables, blood chemistry, blood count, initiation and termination dates of invasive mechanical ventilation (IMV), days in hospital, initial mechanical-ventilator settings, early use (in the first 24 hours after admission) of vasoactive drugs and outcomes.

### Acute kidney injury

AKI staging was based on serum creatinine (sCr) levels. The urine output criterion was not used for diagnosis of AKI since nursing records were out of reach, in COVID-19 areas. The baseline sCr level was defined as the minimum inpatient value during the first 7 days of admission [12]. Diagnosis of AKI was based on the Kidney Disease Improving Global Outcomes (KDIGO) criteria [13]. AKI stage 1 corresponded to an increase in sCr by ≥ 0.3 mg/dL within 48 hours or increase in sCr 1.5 to 1.9 times baseline within the prior 7 days; AKI stage 2 corresponded to an increase in sCr of 2.0–2.9 times baseline; and AKI stage 3 corresponded to an increase in sCr of ≥3 times baseline or initiation of renal replacement therapy (RRT).

### Furosemide stress test

Patients with AKI underwent the furosemide stress test (FST) for prediction of AKI severity. They were euvolemic before undertaking the furosemide challenge. The test was performed by administrating 1 mg/kg of furosemide i.v. or 1.5 mg/kg if the patient had received furosemide within the preceding 7 days, followed by observation of the urinary output in the first 2 hours. The result was considered positive if the patient urinated more than 200 ml per hour, in the following 2 hours after furosemide administration [14].

### Statistical analysis

We performed descriptive statistics including means and standard deviations for normally distributed continuous variables, medians and interquartile ranges for non-parametric distributions, and proportions for categorical variables. Comparisons of the AKI vs. the non-AKI groups were made using Fisher´s exact test for categorical variables and U Mann-Whitey for continuous variables. Comparisons across AKI stages were made using Kruskal-Wallis rank sum test. Logistic regression analysis was used to identify the association between relevant covariates with AKI and mortality. No violations of the assumptions were detected. The multivariate models were built using a stepwise procedure, and variables were entered into the models when the alpha level of risk factor was <0.15 in the univariate analysis. All statistical tests were two-sided, and a P value <0.05 was considered statistically significant.

We also performed the Kaplan-Meier survival analyses for the time to death, comparing the group with AKI vs. the non-AKI group. Statistical analyses were performed using R version 3.6.3. Additionally, a Receiver Operating Characteristic (ROC) curve was constructed in order to define the optimal cut-off points of the variables for prediction to AKI stage 3. Sensitivity, specificity, and the area under the curve (AUC) were calculated with 95% confidence intervals and p values <0.05.

## Results

### Characteristics of study population

**Fig 1:**
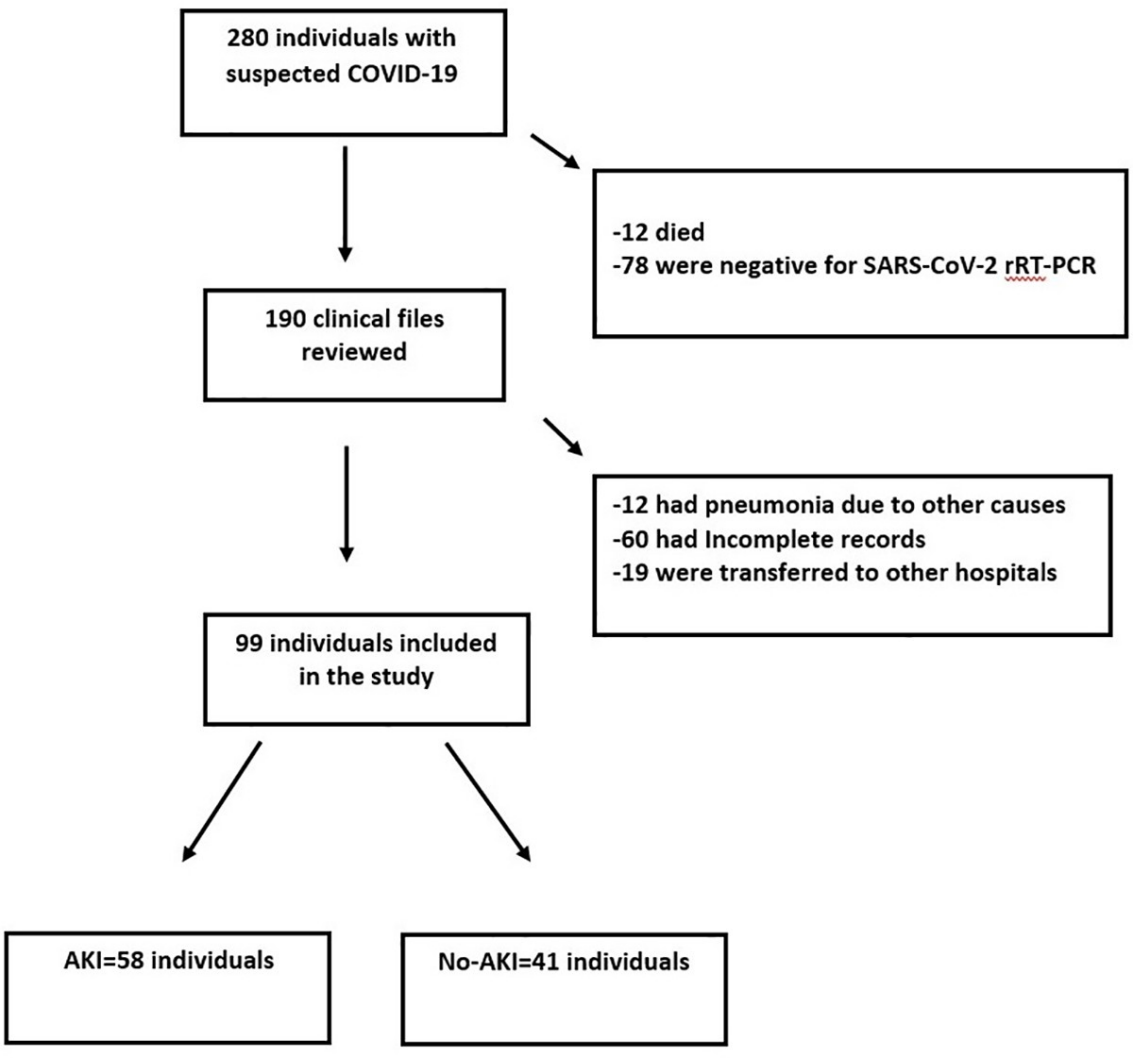
Study diagram. Numbers of individuals assessed for eligibility and individuals included in the study.

During the period between March 1^st^, 2020 and April 30, 2020, a total of 280 individuals were admitted at the INER due to suspected COVID-19. Of those, 12 died during the first 48 hours and 78 had a negative result for the SARS-CoV-2 rRT-PCR test. Therefore, we reviewed the clinical files of 190 individuals. Of those, 12 had pneumonia due to other causes; 19 were transferred to other hospitals due to local saturation; and 60 had incomplete clinical files. Thirty-six patients of the group with incomplete clinical files were not hospitalized because they had a ratio of PaO_2_/FiO_2_ > 300 mm Hg, with acceptable metabolic control (glycemic, kidney and hepatic functions). Therefore, they were trained on the use of supplemental oxygen, the recognition of worsening symptoms, and were monitored from home. The clinical files of the remaining 24 patients were unavailable due to the accelerated reconversion of our institution for exclusive treatment of patients with COVID-19. From the electronic records, we confirmed that five of those patients died and none of them developed AKI during the observation period at hospital. We thus included 99 individuals in the study (Fig 1).

Of the 99 patients included, 74 were male (74.7%); the median age was 52.9 years (SD±13.27); 30 had hypertension (29.7%); 27 had diabetes (26.7%); and 56 had obesity (55.4%; Table 1).

**Table 1:** Baseline characteristics of the study population.

Variables	Overall N = 99	Non-AKI N = 41	AKI N = 58	AKI stage 1 N = 12	AKI stage 2 N = 16	AKI stage 3 N = 29	p value Non-AKI vs. AKI	p value across AKI stages
Age, years^**‡**^	52.9 (13.2)	48.4 (12.3)	56.1(13.2)	57.08 (11.9)	57.25 (13.3)	55.45 (14.1)	**0.01**	0.67
Male [n (%)]	74 (74.7)	30 (73)	44 (73)	9 (75)	12 (75)	22 (75.8)	0.81	1.00
**Symptoms**								
Diarrhea [n (%)]	19 (18.8)	8 (19)	11 (18.9)	2 (16)	4 (25)	5 (17)	1.00	0.79
Rhinorrhea [n (%)]	36 (35.6)	15 (36.5)	21 (36.2)	2 (16.6)	3 (18.7)	15 (51)	1.00	**0.02**
Anosmia [n (%)]	4 (3.96)	2 (4.8)	3 (5.1)	0	0	3 (10.3)	1.00	0.22
Cough [n (%)]	4 (43.96)	34 (82.9)	54 (93.1)	9 (75)	16 (100)	28 (96.5)	0.19	**0.02**
Dyspnea [n (%)]	90 (89.1)	33 (80.4)	57 (98.2)	11 (91.6)	16 (100)	29 (100)	**0.01**	0.15
Odynophagia [n (%)]	53 (52.4)	26 (63.4)	27 (46.5)	4 (33.3)	9 (56.2)	13 (44.8)	0.10	0.48
Fever [n (%)]	93 (92.07)	38 (92.6)	55 (94.8)	11 (91.6)	15 (93.7)	28 (96.5)	0.68	0.80
Headache [n (%)]	93 (92.0)	31 (75.6)	42 (72.4)	9 (75)	12 (75)	20 (68.9)	0.81	0.88
Asthenia [n (%)]	87 (86.1)	34 (82.9)	53 (91.3)	11 (91.6)	14 (87.5)	27 (93.1)	0.22	0.83
Myalgia [n (%)]	84 (83.1)	36 (87.8)	48 (82.7)	10 (83.3)	13 (81.2)	24 (82.7)	0.57	0.98
Expectoration [n (%)]	29 (28.7)	10 (24.3)	19 (32.7)	4 (33.3)	5 (31.2)	10 (34.4)	0.50	0.97
Nausea [n (%)]	15 (14.8)	6 (14.6)	9 (15.5)	2 (16.6)	1 (6.2)	6 (20)	1.00	0.50
**Comorbidities**								
Hypertension [n (%)]	30 (29.7)	9 (21.9)	21 (36.2)	6 (50)	4 (25)	11 (37.9)	0.18	0.39
ACE2 i [n (%)]	4 (3.9)	1 (2.4)	3 (5.1)	0	1 (6.2)	2 (6.8)	0.63	0.65
ARB [n (%)]	15 (14.8)	6 (14.6)	9 (15.5)	4 (33.3)	1 (6.2)	4 (13.7)	1.00	0.17
Obesity [n (%)]	56 (55.4)	10 (25.6)	28 (50.9)	3 (27.2)	6 (37.5)	18 (66.6)	**0.01**	**0.04**
Class 1 [n (%)]	25 (62.5)	7 (70)	18 (60)	3 (75)	3 (50)	11 (57.8)	0.69	0.52
Class 2 [n (%)]	8 (20)	2 (20)	6 (20)	1 (25)	1 (16.6)	4 (21)		
Class 3 [n (%)]	7 (17.5)	1 (10)	6 (20)	0	2 (33.3)	4 (21.0)		
BMI, kg/m^2^ *	29.7 (25.99–33.118)	27.6 (25.1–30.1)	31.1 (27.7–34.7)	28.7 (26.3–31.8)	29.7 (26.2–31.9	32.8 (29.3–37.5)	**0.01**	**0.05**
Diabetes [n (%)]	27 (26.7)	10 (19.6)	17 (34.6)	4 (36.3)	3 (23.0)	10 (40)	0.11	0.58
Dyslipidemia [n (%)]	7 (6.9)	2 (4.8)	5 (8.6)	3 (25)	0	2 (6.8)	0.69	0.06
Heart Disease [n (%)]	4 (3.9)	1 (2.4)	3 (5.1)	1 (9.1)	0	2 (8)	0.35	0.56
Pulmonary Disease [n (%)]	5 (4.95)	2 (3.9)	3 (6)	1 (9.09)	0	2 (8)	0.67	0.56
Rheumatic Disease [n (%)]	3 (2.9)	1 (1.9)	2 (4)	2 (18.1)	0	0	0.61	**0.02**
Allergic [n (%)]	10 (9.9)	5 (9.8)	5 (10.2)	2 (18.1)	0	3 (12)	1.00	0.31
Cancer [n (%)]	3 (2.9)	1 (1.9)	2 (4)	1 (9.09)	0	1 (4)	0.61	0.53
Smoker [n (%)]	3 (2.9)	13 (25.4)	7 (14.2)	3 (27.2)	0	4 (16)	0.21	0.16
Comorbidities [n (%)]		2 (1–2)	2 (1–3)	2 (1–4)	1 (1–1)	2 (1–3)	0.45	**0.01**
**Treatment**								
Drugs before admission [n (%)]	67 (66.3)	34 (66.6)	34 (69.3)	7 (63.6)	8 (61.5)	19 (76)	0.83	0.59
Quinines [n (%)]	81 (80.1)	43 (97.7)	38 (95)	10 (100)	10 (90.9)	18 (94.7)	0.60	0.63
Hydroxychloroquine [n (%)]	43 (42.5)	22 (43.1)	16 (32)	6 (66.6)	5 (50)	10 (55.5)	0.62	0.90
Chloroquine [n (%)]	38 (37.6)	21 (41.1)	19 (38)	3 (33.3)	5 (50)	8 (44.4)		
Oseltamivir [n (%)]	76 (75.24)	37 (72)	39 (79)	10 (100)	11 (100)	18 (94.7)	0.05	0.57
Antibiotics [n (%)]	79 (78.2)	40 (78.4)	39 (79.5)	10 (25.6)	10 (25.6)	19 (48.7)	0.38	0.42
Ceftriaxone [n (%)]	79 (78.2)	38 (74.5)	36 (73.4)	10 (100)	9 (90)	17 (84.2)		
Meropenem [n (%)]	74 (73.2)	1 (1.9)	2 (4)	0	1 (10)	1 (5.2)		
Amikacin [n (%)]	3 (2.9)	0	1 (2)	0	0	1 (5.2)		
Piperacillin/Tazobactam [n (%)]	1 (0.99)	1 (1.9)	0	0	0	1 (5.2)		
Enoxaparin [n (%)]	81 (80.2)	44 (86.2)	37 (75.5)	9 (90)	10 (100)	18 (94.7)	0.21	0.60

AKI, acute kidney injury; ACE2 i, angiotensin converting enzyme inhibitor (enalapril, captopril); ARB, angiotensin II receptor blocker (losartan, telmisartan); BMI, body mass index; Class 1 obesity: BMI of 30 to < 35 kg/m^2^; Class 2 obesity: BMI of 35 to < 40 kg/m^2^; Class 3 obesity: BMI of 40 kg/m^2^ or higher.

*Data are expressed as medians (interquartile ranges).

^**‡**^Data are expressed as means (standard deviation).

### Acute kidney injury

**Fig 2:**
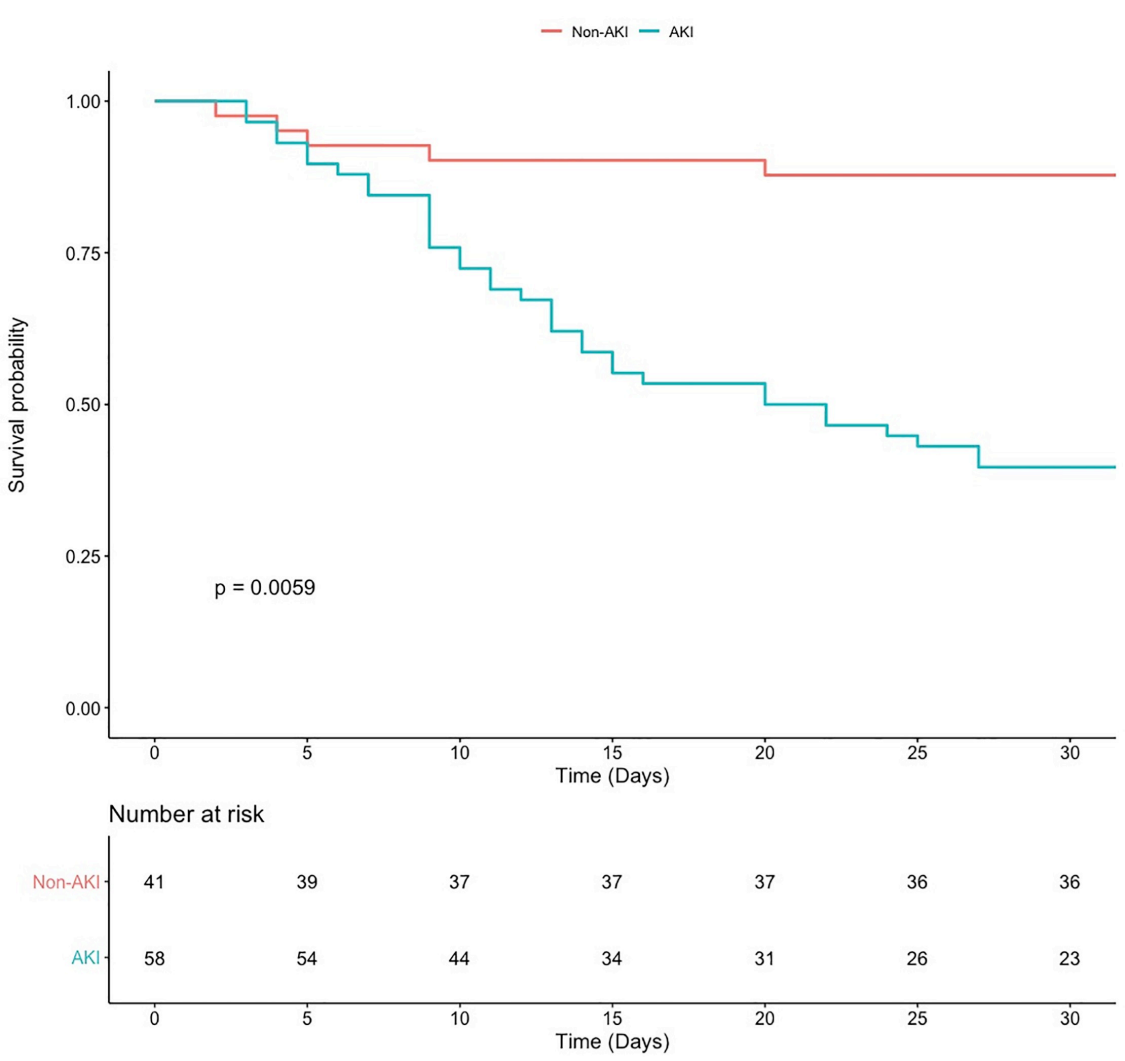
Kaplan-Meier survival curves. Time to death for the AKI group (blue line), and the non-AKI group (red line) during a follow-up period of 30 days. Time 0 corresponded to hospital admission.

Fifty-eight patients developed AKI (the AKI group) and 41 individuals did not develop AKI (the non-AKI group, Table 1). Of those, 12 had AKI stage 1 (21.1%); 16 had AKI stage 2 (28.1%); and 29 had AKI stage 3 (50.9%). Forty-one patients of the AKI group (83.6%) required IMV, while 23 patients of the non-AKI group (45%) required IMV (p = 0.01). Twenty-six patients of the AKI group required prone mechanical ventilation (57.7%), compared with 2 patients (14%) in the non-AKI group (p = 0.01). On admission, 31 patients of the AKI group required vasoactive drugs (63.2%), compared with 18 patients (35.2%) of the non-AKI group (p = 0.01). Survival curves of both groups decreased with a similar rate on the first days in hospital, but since day 10 this decrease was steeper in the AKI group. By day 30, in-hospital mortality was significantly higher in the AKI group (38 patients in the AKI group (65.5%) vs. 6 patients (14.6%) in the non-AKI-group, p = 0.001; Fig 2).

In-hospital mortality was significantly higher in patients with AKI stage 3 (79.3%) and AKI stage 2 (68.7%) compared with those with AKI stage 1 (25%; p = 0.01). The body mass index (BMI) was significantly higher in the AKI group (31.1 kg/m^2^ vs. 27.6 kg/m^2^ in the non-AKI group; p = 0.01). Obesity was more frequent in the AKI group (28 patients, 50.9%) than in the non-AKI group (10 patients, 25.6%; p = 0.01). The median time to AKI development was 6.5 days (SD±8.33).

**Fig 3:**
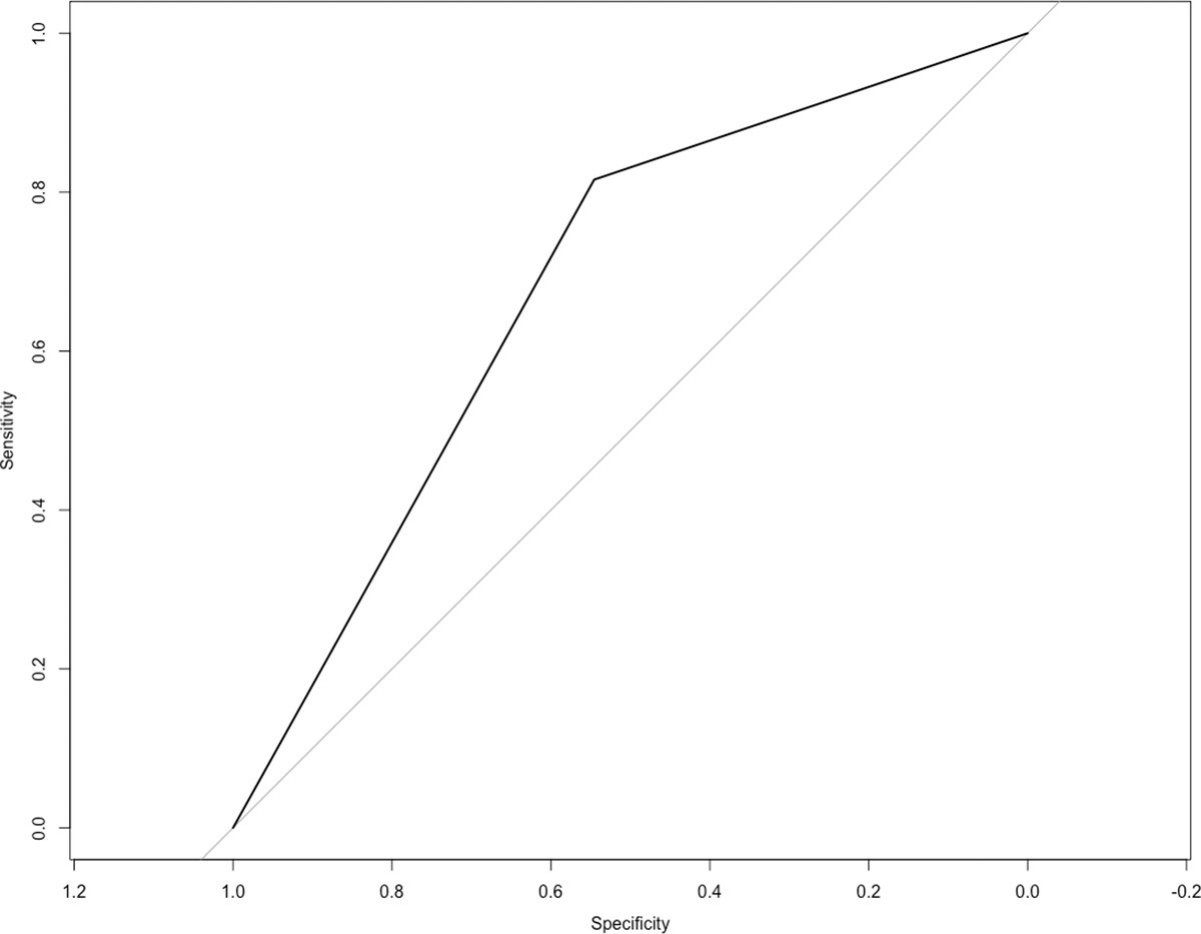
Receiver-operating characteristic (ROC) curve for the furosemide stress test (FST). A ROC curve was constructed to evaluate FST performance in predicting AKI stage 3 development in 53 patients with COVID-19. AUC = 0.681 (95% CI = 0.514–0.847), p = 0.009, sensitivity = 81.6%, specificity = 54.5%.

Fifty-three patients underwent the furosemide stress test. Of those, 39 had a positive result (73.5%) and 14 had a negative result (26.4%). Of the 53 patients undergoing the FST, 12 progressed to AKI stage 3 (22%). The ROC curve for the FST had an AUC of 0.681 (95% confidence interval (CI) = 0.514–0.847, p = 0.009), with a sensitivity of 81.6% and a specificity of 54.5% (Fig 3).

A total of 11 patients (22.4%) required renal replacement therapy (RRT). Of those, 5 used continuous RRT, 3 used intermittent hemodialysis (IHD), and 3 used prolonged intermittent renal replacement therapies (PIRRT). The median time to RRT initiation after AKI initiation was 4.18 days (SD±3.8) and median time under RRT was 4.29 days (SD± 2.82). Five patients died after 30 days of follow-up, 2 of them had been discharged due to clinical improvement and 3 were hospitalized.

### Inflammation markers and AKI

Some inflammation markers were higher in the AKI group, including C-reactive protein: 19.57 (±9.5) mg/dl in the AKI group vs. 15.03 (±9.3) mg/dl in the non-AKI group, p = 0.03; D-dimer: 1.5 μg/ml (interquartile range (IQR), 0.83–2.26) in the AKI group vs. 0.84 μg/ml (IQR, 0.6–1.29) in the non-AKI group, p = 0.01; procalcitonin: 0.25 ng/ml (IQR, 0.11–0.71) in the AKI group vs 0.18 ng/ml (IQR, 0.08–0.3) in the non-AKI group, p = 0.01; and troponin: 8 pg/ml (IQR, 4.25–38.6) in the AKI group vs. 3 pg/ml (IQR, 2–5.2) in the non-AKI group, p = 0.01. Lymphocytes were decreased in the AKI group: 0.7 10x^3^ mm^3^ (IQR, 0.5–1) compared to the non-AKI group: 0.9 10x^3^ mm^3^ (IQR, 0.8–1.33, p = 0.01; Table 2).

**Table 2:** Baseline laboratory values of the study population.

Variables	Overall N = 99	Non-AKI N = 41	AKI N = 58	AKI stage 1 N = 12	AKI stage 2 N = 16	AKI stage 3 N = 29	p value Non-AKI vs. AKI	p value across AKI stages
**Critical Care Variables**								
IMV [n (%)]	65 (64.3)	23 (45)	41 (83.6)	8 (72.7)	12 (92.3)	21 (84)	**0.01**	0.44
Prone MV [n (%)]	28 (47.4)	2 (14)	26 (57.7)	6 (75)	11 (84)	9 (39)	**0.01**	**0.01**
Vasoactive Drugs [n (%)]	49 (48.5)	18 (35.2)	31(63.2)	6 (75)	8 (72.7)	17 (80.9)	**0.01**	0.85
**Laboratory studies**								
Creatinine, mg/dL*	0.98 (0.84–1.51)	0.87 (0.74–0.99)	1.23 (0.9–1.72)	1.02 (0.91–1.56)	1.13 (0.92–1.5)	1.51 (0.89–1.9)	**0.01**	0.55
Nitrogen urea mg/dL*	18.11 (13.9–26.2)	14.2 (10.9–20.24)	23 (16–36.5)	22.1 (17.0–26.6)	19.05 (15.5–23.8)	28.7 (16.1–37.03)	**0.01**	0.26
Creatinine mg/dL, day 7*	1.04 (0.71–1.58)	0.72 (0.62–0.91)	1.4 (1–1.92)	1.19 (0.77–1.47)	1.32 (0.93–1.84)	1.68 (1.12–2.57)	**0.01**	0.06
Creatinine, mg/dL on AKI*	1.62 (1.37–2.06)	NA	1.62 (1.37–2.06)	1.31 (1.1–1.48)	1.52 (1.35–1.79)	1.93 (1.62–2.99)	NA	**0.01**
Maximum Creatinine, mg/dL*	2.5 (1.71–5.36)	NA	2.5 (1.71–5.36)	1.4 (1.18–1.59)	1.87 (1.56–2.3)	5.5 (2.99–6.63)	NA	**0.01**
CPK, U/I*	98.8 (60–193.3)	89.4 (50.4–268.62	108.8 (69.1–177.1)	139.5 (103.0–311.7)	107.7 (64.7–168.4)	89 (71.2–174.9)	0.55	0.38
Glucose, mg/dL*	111.6 (98.2–153)	101.3 (90.5–111.9)	124.7 (108.8–184.2)	127.9 (119.2–237.6)	112.15 (102–129.4)	136.5 (110.2–194.7)	**0.01**	0.07
Uric acid, mg/dL*	5.17 (4.07–7.58)	4.52 (3.6–5.9)	5.7 (4.6–7.3)	6.3 (5.06–6.9)	4.9 (3.5–6.7)	6.27 (4.73–7.58)	**0.01**	0.26
PaO_2_/FiO_2_, mm/Hg*	194.05 (123.14–238.81)	211.67 (153–248)	181.19 (121–228)	212.5 (146–213)	194 (146–224)	172.8 (108–224)	0.12	0.76
Lactate dehydrogenase, UI/L*	373.1 (235–512.3)	346.45 (291.3–492.4)	378 (208–519.7)	369.9 (263.5–643.3)	398.2 (296–453.1)	390.8 (214.6–535.4)	0.56	0.97
C-reactive protein, mg/dL^**‡**^	17.72 (9.65)	15.03 (9.3)	19.57 (9.5)	21.5 (9.7)	20.9 (6.6)	16.8(8.7)	**0.03**	0.09
D-dimer, μg/ml*	1.1 (0.7–1.78)	0.84 (0.6–1.29)	1.5 (0.83–2.26)	1.09 (0.79–1.8)	1.43 (0.88–2.34)	1.75 (0.97–2.32)	**0.01**	0.32
Procalcitonin, ng/ml*	0.2 (0.09–0.58)	0.18 (0.08–0.3)	0.25 (0.11–0.71)	0.22 (0.14–0.67)	0.26 (0.19–0.46)	0.24 (0.1–1.2)	**0.01**	0.85
Troponin, pg/ml*	4.8 (2.5–12.9)	3 (2–5.2)	8 (4.25–38.6)	8.1 (2.1–55.1)	12 (7.55–14.8)	6.05 (4.35–51.2)	**0.01**	0.82
Albumin, g/dL^**‡**^	3.44 (0.5)	3.63 (0.5)	3.3 (0.46)	3.56 (0.3)	3.12 (0.38)	3.28 (0.52)	**0.01**	**0.02**
Hemoglobin, g/dL*	15.3 (14.4–16.3)	15.2 (14.6–15.8)	15.3 (14.4–16.6)	16.1 (15.1–16.7)	14.7 (13.3–16.03)	15.4 (14.8–16.2)	0.48	0.23
Leucocytes, 10x^3^ mm^3^*	8.9 (6.6–11.5)	8.05 (6.5–11)	9 (6.7–11.5)	9.45 (6.3–11.5)	7.3 (6.38–9.2)	9.5 (7.25–12.5)	0.34	0.21
Lymphocytes, 10x^3^ mm^3^*	0.8 (0.6–1.2)	0.9 (0.8–1.33)	0.7 (0.5–1)	0.7 (0.48–0.72)	0.7 (0.5–1.02)	0.85 (0.5–1.2)	**0.01**	0.194
Platelets 10x^3^mm^3^*	202 (171–266)	217.5 (178.5–266.75)	201 (162–257)	196.5 (158.25–251.75)	186 (165.5–267.2)	201.5 (165–261.25)	0.34	0.85
Platelets/Lymphocytes Index*	246.25 (173.64–340)	233.75 (162–264)	300 (202.2–424)	317.4 (267.7–460)	308.1 (234.5–419.9)	282.7 (176.6–383.4)	**0.01**	0.45
Platelets/Neutrophils Index*	29.13 (20.49–41.19)	34.9 (25.08–44.91)	27.66 (19–36.5)	27.1 (19.3–33.04)	28.8(25.4–39.5)	26.1 (18.8–36.5)	**0.02**	0.56
**Outcomes**								
Mortality (%)*	44 (44.4)	6 (14.6)	38 (65.5)	3 (25)	11 (68.7)	23 (79.3)	**0.01**	**0.01**
Days in hospital*	12 (8.5–23.5)	9 (8–16)	14 (9–30)	14.5 (9.75–32.25)	13.5 (9–24.75)	15(9–33)	**0.02**	0.91
Days to death*	12 (8.5–23.5)	7 (4.25–17.25)	12.5 (9–20)	9 (8–18)	13 (9–15)	12 (8–21)	**0.01**	0.96
Days to AKI*	2 (1–9)	NA	2 (1–9)	3 (1–9)	4 (1–10.75)	2 (1–8)	NA	0.77
Days on IMV*	1 (0–1)	1 (0–1)	1 (0–1)	1 (0–1)	1(0–1)	1(0–1)	0.96	0.80

AKI, acute kidney injury; CPK, creatine phosphokinase; PaO_2_/FiO_2_ arterial partial pressure of oxygen/fraction inspired oxygen; IMV, invasive mechanical ventilation; Prone MV, prone mechanical ventilation; vasoactive drugs (norepinephrine and vasopressin on admission). Admission laboratory test results were used unless otherwise specified.

*Data are expressed as medians (interquartile ranges).

^**‡**^Data are expressed as means (standard deviation).

On admission, 82.8% had a positive result for SARS-CoV-2-rRT-PCR, but all patients had a positive result when the test was performed for the third time. Sixty-five patients (64.3%) required IMV. The median positive end-expiratory pressure (PEEP) level was 11.5 cm H_2_O (SD ±2.4); and 28 required prone ventilation due to refractory hypoxemia. Forty-nine patients (48.5%) required vasoactive drugs on admission; and overall mortality was 44.4%. All patients had ground glass opacities; 85.2% had crazy paving pattern; 94.3% had consolidation; 5.6% had pleural effusion; 79.5% had bronchiectasis; 54.5% had atelectasis; 59% had peripheral distribution; and 39.7% had central and peripheral distribution.

### Risk factors for acute kidney injury

The univariate analysis indicated that patients with AKI were older (unadjusted odds ratio (OR) = 1.05, 95% CI = 1.01–1.08, p = 0.007); had a higher BMI (OR = 1.10, CI = 1.02–1.18, p = 0.012); a higher frequency of obesity (OR = 2.59, 95% CI = 1.11–6.04, p = 0.028); a higher requirement of IMV (OR = 6.78, 95% CI = 2.69–17.04, p = 0.001); a higher requirement of vasoactive drugs (OR = 4.26, CI = 1.08–10.06, p = 0.001); a higher ratio of platelet/lymphocyte (OR = 1.0037, CI = 1.0005–1.0068, p = 0.021); a lower count of lymphocytes (OR = 0.23, CI = 0.08–0.67, p = 0.007); a higher level of C-reactive protein (OR = 1.05, CI = 1.01–1.1, p = 0.028); and a lower level of albumin (OR = 0.26, CI = 0.11–0.64, p = 0.003). After adjusting for possible confounding variables, the multivariate analysis indicated that the risk factors for AKI were older age (OR = 1.07, 95% CI = 1.01–1.13, p = 0.024); obesity (OR = 6.58, 95% CI = 1.8–24.05, p = 0.040); and requirement of IMV (OR = 6.18, CI = 1.29–29.58, p = 0.023, Table 3).

**Table 3:** Risk factors for acute kidney injury.

Variables	Unadjusted OR (95% CI)	p value	Adjusted OR (95% CI)*	p value
Age, years	1.05 (1.01–1.08)	**0.007**	1.07 (1.01–1.13)	**0.024**
Male	1.15 (0.46–2.88)	0.762	0.88 (0.24–3.21)	0.849
BMI	1.10 (1.02–1.18)	**0.012**	-	-
Obesity (BMI ≥ 30 kg/m^2^)	2.59 (1.11–6.04)	**0.028**	6.58 (1.8–24.05)	**0.040**
Hypertension	2.02 (0.81–5.03)	0.132	0.25 (0.05–1.15)	0.750
Diabetes	2.56 (0.96–6.79)	0.060	1.91 (0.05–7.3)	0.342
PaO_2_/FIO_2_, mmHg	0.99 (0.99–1.00)	0.302	-	-
IMV	6.78 (2.69–17.04)	**0.001**	6.18 (1.29–29.58)	**0.023**
Vasoactive drugs	4.26 (1.08–10.06)	**0.001**	1.54 (0.37–6.43)	0.551
Platelet/Lymphocyte ratio	1.0037 (1.0005–1.0068)	**0.021**	1.0033 (0.9983–1.0084)	0.198
Lymphocytes 10x^3^ mm^3^	0.23 (0.08–0.67)	**0.007**	0.69 (0.12–3.83)	0.670
Procalcitonin, ng/ml	1.1 (0.93–1.32)	0.259	-	-
D-dimer, μg/ml	1.29 (0.95–1.75)	0.102	1.23 (0.89–1.71)	0.202
Troponin, pg/ml	1.00 (0.99–1.001)	0.984	-	-
C-reactive protein, mg/dL	1.05 (1.01–1.1)	**0.028**	0.9984 (0.9366–1.0642)	0.961
Albumin g/dL	0.26 (0.11–0.64)	**0.003**	1.24 (0.29–5.28)	0.769

OR, odds ratio; CI, confidence interval; BMI, body mass Index; IMV, invasive mechanical ventilation; PaO_2/_FiO_2_, arterial partial pressure of oxygen/fraction inspired oxygen ratio; AKI, acute kidney injury; vasoactive drugs (norepinephrine and vasopressin on admission).

Admission laboratory test results were used.

* Variables were entered into the model when the alpha level of risk factor was less than 0.15. Age and gender were added into the model regardless of alpha level.

#### Risk factors for mortality

The univariate analysis indicated that deceased patients had a higher BMI (OR = 1.14, 95% CI = 1.06–1.23, p = 0.001); a higher frequency of obesity (OR = 3.6, 95% CI = 1.56–8.32, p = 0.003); a higher requirement of IMV (OR = 7.47, IC = 2.71–20.57, p = 0.001) and of vasoactive drugs (OR = 7.18, IC = 2.95–17.46, p = 0.001); a higher level of C-reactive protein (OR = 1.06, CI = 1.02–1.11, p = 0.008); a lower level of albumin (OR = 0.25, IC = 0.1–0.62, p = 0.003); and a higher frequency of AKI (OR = 12.96; IC = 4.63–36.28, p = 0.001). After adjusting for possible confounding variables, the multivariate analysis indicated that the risk factors for mortality were obesity (OR = 5.57, 95% CI = 1.48–20.93, p = 0.011); requirement of vasoactive drugs on admission (OR = 5.35, 95% CI = 1.16–24.61, p = 0.031); and AKI (OR = 8.61, 95% CI = 2.24–33.1, p = 0.002, Table 4).

**Table 4:** Risk factors for mortality.

Variables	Unadjusted OR (95% CI)	p value	Adjusted OR (95% CI)*	p value
Age, years	1.02 (0.99–1.06)	0.119	1.009 (0.9457–1.0593)	0.975
Male	1.42 (0.06–3.57)	0.455		
BMI	1.14 (1.06–1.23)	**0.001**		
Obesity (BMI ≥ 30 kg/m^2^)	3.6 (1.56–8.32)	**0.003**	5.57 (1.48–20.93)	**0.011**
Hypertension	1.8 (0.76–4.29)	0.182		
Diabetes	1.65 (0.68–4.03)	0.269		
Comorbidities				
PaO_2_/FIO_2_, mmHg	0.9963 (0.9912–1.0014)	0.152		
IMV	7.47 (2.71–20.57)	**0.001**	1.4 (0.22–8.77)	0.720
Vasoactive drugs	7.18 (2.95–17.46)	**0.001**	5.35 (1.16–24.61)	**0.031**
Platelet/Lymphocyte ratio	1.0013 (0.999–1.0036)	0.274		
Lymphocytes 10x^3^ mm^3^	0.54 (0.2–1.44)	0.217		
Procalcitonin, ng/ml	1.0068 (0.9749–1.0397)	0.680		
D-dimer, μg/ml	1.17 (0.97–1.41)	0.100	1.04 (0.84–1.29)	0.723
Troponin, pg/ml	1.01 (1–1.02)	0.118	1.0062 (0.9962–1.0163)	0.228
C-reactive protein, mg/dL	1.06 (1.02–1.11)	**0.008**	1.02 (0.95–1.08)	0.653
Albumin g/dl	0.25 (0.1–0.62)	**0.003**	0.83 (0.2–3.51)	0.802
AKI	12.96 (4.63–36.28)	**0.001**	8.61 (2.24–33.1)	**0.002**

OR, odds ratio; CI, confidence interval; BMI, body mass Index; IMV, invasive mechanical ventilation; PaO_2/_FiO_2_, arterial partial pressure of oxygen/fraction inspired oxygen ratio; AKI, acute kidney injury; vasoactive drugs (norepinephrine and vasopressin on admission).

Admission laboratory test results were used.

* Variables were entered into the model when the alpha level of risk factor was less than 0.15. Age and gender were added into the model regardless of alpha level.

## Discussion

Multiple organ involvement including the liver, gastrointestinal tract and kidney have been reported in patients with COVID-19 [8]. Since information about causes leading to severe kidney disease in these patients is still limited, here we determined the factors associated with the development of AKI and explored the relation between AKI and mortality in Mexican population with severe COVID-19.

In our cohort of patients with severe COVID-19, the risk factors for AKI were older age, obesity and requirement of IMV on admission. The risk factors for mortality were obesity, requirement of vasoactive drugs on admission and AKI. Moreover, in-hospital mortality was particularly elevated in patients with AKI stages 2 and 3.

In contrast with initial studies reporting low incidences of AKI between 5–7% in hospitalized patients with COVID-19 in China [8, 15, 16], the incidence of AKI in our cohort was 58.6%, and half of those had severe AKI (stage 3). Differences between studies might be partially explained by the fact that our institution is a national referral center for respiratory diseases, where mostly patients with COVID-19 severe disease are being admitted. Our study population was similar to that studied in another Mexican, third-level, national referral center for patients with severe COVID-19, reporting an AKI incidence of 60.7% [17]; and to the cohort studied in a New York City medical center, where up to 78% of the patients developed AKI and most of them required IMV [18].

As previously reported, we found that older age was a risk factor for AKI in hospitalized patients with COVID-19 [9]. Obesity was a risk factor for AKI and mortality in our cohort, and it has been reported as a common comorbidity in hospitalized patients with COVID-19 [19, 20], and as a risk factor for hospital admission and need for critical care [21]. This is particularly relevant for countries with high obesity rates, such as Mexico. In the adult Mexican population, the combined prevalence of overweight and obesity is approximately 71% [22]. It has been suggested that chronic inflammation in obesity is apparent, with an increased level of interleukin-6, adipokines and pro-inflammatory cytokines (e.g., TNF-alpha, interferon), inducing a chronic low-grade inflammatory state and impairing immune response [23, 24]. A possible mechanism related to COVID-19 severity in obese persons is speculated to occur through a functional restrictive capacity of the obese lung. It would also be interesting to understand whether the obese patients had higher PEEP or driving pressure that could account for the higher risk for AKI. Unfortunately, PEEP data retrieval was incomplete in our retrospective review of medical records, so we were unable to perform this analysis. The need for mechanical ventilation on admission was also a risk factor for AKI in our cohort. It is well known that the main causes of AKI are hypoxia, ischemia and nephrotoxicity. The kidney is particularly susceptible to ischemia and toxins, resulting in vasoconstriction, endothelial damage, and activation of inflammatory processes [25]. In hospitalized patients with severe COVID-19, the important relationship between AKI and respiratory failure was previously reported [9].

The requirement of vasoactive drugs on admission was a risk factor for mortality in our cohort. This is not surprising if we consider that vasoactive drugs are used in the most critically ill patients with septic shock and evidence of renal dysfunction [26]. In this context, early start of vasopressor support is aimed at having a more rapid restoration of blood flow in combination with lower fluid accumulation, allowing early restitution of tissue perfusion while avoiding fluid overload-mediated harm [27].

We found that elevated serum creatinine on admission was more common in patients with AKI, which was previously reported in a cohort in China [8]. This means that patients with kidney involvement on admission were more likely to develop AKI. Lymphopenia was more common in the group with AKI, and low lymphocyte counts have been associated with severe COVID-19 and longer hospital stay [28]. COVID-19-associated lymphopenia might derive from retention of lymphocytes in the lung. Also, lymphocytes express the angiotensin-converting enzyme 2 (ACE2) receptor on their surface [29]. Thus, SARS-CoV-2 infection may directly induce lysis of these cells. In addition, elevated levels of pro-inflammatory cytokines, may promote lymphocyte apoptosis.

D-dimer elevation on admission was more common in patients with AKI. This molecule is a product of cross-linked fibrin degradation and is a sensitive marker of thrombosis and coagulation activation [30]. Elevated D-dimer level has been consistently reported in patients with COVID-19 [31, 32], and its gradual increase during disease course is particularly associated with disease worsening [33]. Elevated C-reactive protein on admission was more common in patients with AKI. Higher C-reactive protein has been linked to unfavorable aspects of COVID-19 disease, such as ARDS development [34], higher troponin-T levels and myocardial injury [35], and death [36].

Troponin levels on admission were higher in the group with AKI. High-sensitivity cardiac troponin T (hs-cTnT) and cardiac troponin I (cTnI) have been associated with AKI and are useful plasma biomarkers of cardiac injury [37]. In patients with severe COVID-19, elevated troponin level might indicate cardiovascular stress resulting from direct SARS-CoV-2 infection of the heart [38], hemodynamic changes, or underlying cardiac injury and dysfunction [39].

The original study describing the furosemide stress test for prediction of AKI outcome reported an AUC of 0.87 in the first two hours, with a sensitivity of 87.1% and specificity of 84.1% [14]. That study was performed in a heterogeneous population of patients exposed to different nephrotoxic factors (nonsteroidal anti-inflammatory drugs, aminoglycosides, amphotericin, contrast, post-cardiac surgery and sepsis). We found lower values of AUC (0.681), sensitivity (81.6%) and specificity (54.5%). Differences between studies may be partially explained by the fact that we only included patients with sepsis related with SARS-CoV-2 infection. Despite these considerations, we deem that the furosemide stress test is an easy, non-invasive and accessible technique which may contribute to predict the severity of AKI in patients with SARS-CoV-2 infection. Larger, prospective validations of the furosemide stress test in patients with severe COVID-19 are required because improving risk prediction in those with early AKI would be of high value for patient care and clinical decision making.

The main limitation of our study was its retrospective design. Also, the number of patients included in the study was low. Another study limitation was that patients with incomplete clinical files or those who were transferred to other hospitals due to local saturation were not included in the study, and this may represent a selection bias.

Considering that standardized definitions of AKI are based on sCr and urine output [40], then inaccessibility to nursing records restricted to COVID-19 areas represents an important study limitation because urine output was not used for diagnosis of AKI, and sCr was not adjusted for fluid-balance. The lack of pre-hospital baseline sCr measurements was also a study limitation because baseline sCr values were an estimation. Since we could not assess the baseline renal status, we could not explore whether CKD itself is a risk factor for AKI in the context of COVID-19. One additional study limitation is that we retrieved information during hospitalization, but a longer observation period would have provided additional information regarding the clinical outcome and the impact of AKI in the population studied. That is, we were not able to report the proportion of individuals developing CKD in the group with AKI. Finally, our study was conducted at a national referral center for respiratory diseases receiving disproportionately more patients with severe COVID-19, and this represents a potential source of referral bias. Nevertheless, this particular cohort was suitable for identifying the risk factors for AKI in Mexican individuals with severe COVID-19.

## Conclusions

AKI was common in our cohort of patients with severe pneumonia caused by SARS-CoV-2 infection. The risk factors for AKI were older age, obesity and requirement of IMV on admission. The risk factors for mortality were obesity, requirement of vasoactive drugs on admission and AKI. Mortality was more frequent in patients with AKI stages 2–3. The FST had an acceptable predictive capacity to identify patients progressing to AKI stage 3. Still, larger, prospective validations of the FST in patients with severe COVID-19 are required.

## Supporting information

S1 File. Raw data This file includes the minimal anonymized data set necessary to replicate our study findings. (XLSX)
